# Mutational induction in SARS-CoV-2 major lineages by experimental exposure to neutralising sera

**DOI:** 10.1038/s41598-022-16533-4

**Published:** 2022-07-21

**Authors:** Martina Brandolini, Giorgio Dirani, Francesca Taddei, Silvia Zannoli, Agnese Denicolò, Valentina Arfilli, Arianna Battisti, Martina Manera, Andrea Mancini, Laura Grumiro, Maria Michela Marino, Giulia Gatti, Michela Fantini, Simona Semprini, Vittorio Sambri

**Affiliations:** 1 Unit of Microbiology, The Greater Romagna Area Hub Laboratory, 47522 Cesena, Italy; 2 Department of Experimental, Diagnostic and Specialty Medicine (DIMES)-Alma Mater Studiorum, University of Bologna, 40138 Bologna, Italy

**Keywords:** SARS-CoV-2, Viral genetics

## Abstract

The ongoing evolution of SARS-CoV-2 and the emergence of new viral variants bearing specific escape mutations responsible for immune evasion from antibody neutralisation has required a more accurate characterisation of the immune response as one of the evolutive forces behind viral adaptation to a largely immunised human population. In this work, culturing in the presence of neutralising sera vigorously promoted mutagenesis leading to the acquisition of known escape mutations on the spike as well as new presumptive escape mutations on structural proteins whose role as target of the neutralizing antibody response might have been thus far widely neglected. From this perspective, this study, in addition to tracing the past evolution of the species back to interactions with neutralising antibody immune response, also offers a glimpse into future evolutive scenarios.

## Introduction

After raging through the world for over a year, SARS-CoV-2 has by far shown few signs of abating, proving to possess extraordinary and unprecedented adaptive capacities which have allowed its almost uncontrolled global spread, regardless of the adopted containment measures, accounting for over 250 million confirmed cases and about 5 million attributable deaths from the outbreak of the pandemic to date^1–3^. Paradoxically if we consider the current situation, SARS-CoV-2 shows a much lower variability than other RNA viruses due to the presence, within its replicative machinery, of a proofreading activity (function performed by non-structural protein 14, or nsp14, also referred to as ExoN), which promptly corrects mismatch errors randomly introduced in the genetic material as a natural by-product of genome replication. If, on the one hand, by improving the fidelity of replication, it allows Coronaviruses to extend the size of their genome beyond the theoretical dimensions imposed for RNA viruses, on the other hand, it could also greatly reduce the possibilities of diversification of the genetic material, at least under a theoretical point of view^4,5^. However, this does not seem to have hindered SARS-CoV-2 evolution. The appearance of new variants, either classified as VOI (Variants Of Interest) or VOC (Variants Of Concern), the latter bearing mutations that can increase viral infectivity, reduce effectiveness of diagnostics and therapeutics or, above all, contribute to the evasion from antibody immune response, both developed following a previous infection as much as induced by vaccination, has prompted to better define the evolutionary forces behind SARS-CoV-2 genetic evolution and, above all, delineate the role played by the immune response, in order to predict its evolutionary trajectory in the context of a largely immunised population.

The main target of the neutralising antibody immune response is represented by the spike glycoprotein, which consists of two subunits: S1, containing the receptor binding domain (RBD), responsible for interactions with the cellular receptor ACE2, and S2, responsible for fusion between viral envelope cellular membranes^6,7^. The S1 subunit has two highly immunogenic domains, namely the N-terminal domain and the RBD, which are prone to the accumulation of escape mutations, remarkably abundant in all variants reported to date^8–12^.

To estimate to which extent the antibody immune response has contributed to the evolution of lineage B.1, and therefore to the appearance of new lineages, and the further capacity of the lineages of greatest interest to mutate in response to the selective pressure exerted by neutralising antibodies, these were sequentially passaged in cell culture in the presence of scalar concentrations of neutralising sera. Besides lineage B.1, lineages B.1.1.7, B.1.351, P.1 and B.1.525 (or Alpha, Beta, Gamma and Eta variants, according to the World Health Organisation classification system) were included. Lineage B.1 was cultured separately with low, medium, and high titre sera (hereinafter, for brevity, referred to as P40, P160 and P1280) to estimate what role poorly, moderately, or highly neutralising antibody immune responses may have played in the emergence of new mutations capable of conferring resistance to humoral antibody immunity and consequently in the appearance of the different variants currently in circulation. The other lineages were cultured only with highly neutralising serum, considering their more or less marked resistance to neutralisation, already widely reported in literature^13–15^, in order to evaluate their further evolutionary potential when exposed to neutralising antibodies.

## Results

### Lineage B.1

**Figure 1:**
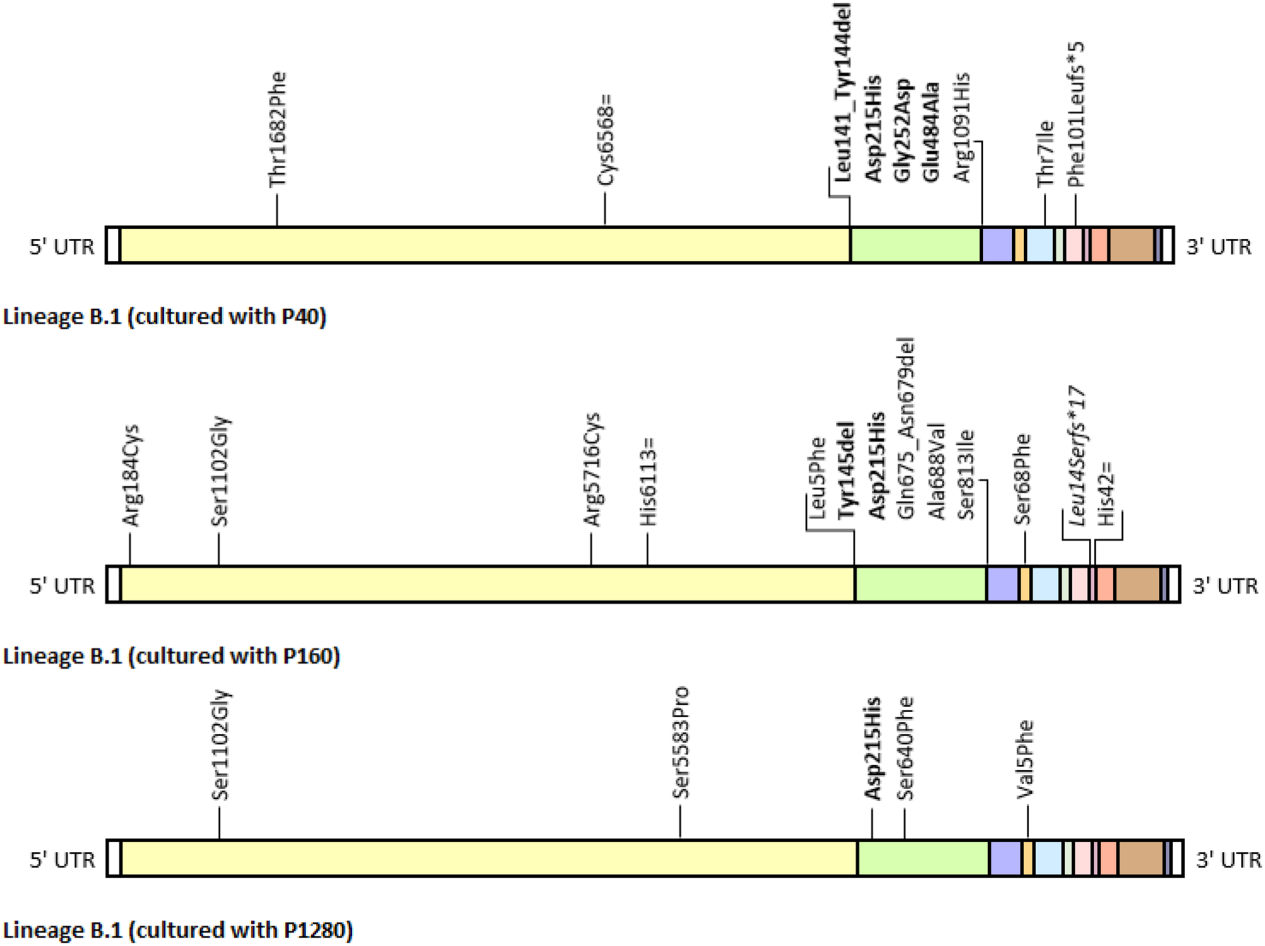
Schematic representation of newly acquired mutations identified in the 90-day isolates of lineage B.1 maintained in culture in the presence of poorly, moderately, and highly neutralising serum pools (P40, P160 and P1280, respectively). Known escape mutations are highlighted in bold, while other presumptive escape mutations are highlighted in italics. The “ = ” symbol indicates a synonym mutation, whereas the “fs*” symbol indicates a frameshift mutation causing the early truncation of the protein at the specified amino acidic position. From 5’ UTR to 3’ UTR (both in white): ORF1ab (yellow), S (green), ORF3a (blue), E (orange), M (light blue), ORF6 (grey), ORF7a (pink), ORF7b (lilac), ORF8 (red), N (brown) and ORF10 (lavender).

Overall, in the three experimental conditions, i.e. in the presence of a low, medium or high selective antibody pressure, lineage B.1 showed a different evolutionary capacity resulting in the accumulation of a more or less considerable number of mutations, which involved genes coding for proteins whose role as targets of the immune response has already been clarified (spike glycoprotein), genes coding for structural proteins (envelope and membrane proteins), whose role in this respect needs to be further investigated, as well as genes encoding non-structural and accessory proteins (ORF1ab, on the one hand, ORF7a and ORF7b, on the other). Culturing in the presence of poorly and moderately neutralising antibodies seems to have most promoted the accumulation of mutations: in both conditions, a total of 20 mutational events were identified over 90 days (including by this term the acquisition of new mutations and the loss of mutations either present in the original isolate or appeared during the study). Considering an estimated mutation rate of 6·10^–4^ mutations/genome/year (CI: 4·10^–4^–7·10^–4^)^16^, roughly corresponding to 23 mutations accumulated in one year (just under 2 per month), the calculated mutation rate for both culture conditions was significantly higher (2.1·10^–3^ m/g/year), highlighting how SARS-CoV-2 has undergone an accelerated evolution under a poor or moderate selective antibody pressure. By comparison, passaging in the presence of highly neutralising antibodies only led to 6 mutational events, corresponding to a calculated mutational rate slightly higher than that reported in literature, but notwithstanding within the confidence interval (6.3·10^–4^ m/g/year). Newly acquired mutations identified in the 90-day isolates of lineage B.1 maintained in the three culturing conditions are summarised in Fig. 1.

A considerable number of mutations fell on the spike coding sequence, affecting immunodominant sites (NTD and RBD on S1 subunit) as well as regions without any relevance under this point of view (both on S1 and S2 subunits). Some of the mutations identified on the S gene have already been reported in scientific publications, but for most of them no reference is made to particular onset scenarios or specific phenotypic consequences of relevance as regards infectivity or immune escape, thus suggesting that they are, for the most part, natural polymorphisms, simply resulting from viral replication^17,18^. These comprehend Leu5Phe and Ser640Phe in the N-terminal domain, Ala688Val substitutions and Glu675_Asn679 and Asn679_Arg685 deletions, all adjacent to the furin cleavage site, and, lastly, Ser813Ile and Arg1091His on the S2 subunit. Other mutations identified on the S gene, on the other hand, have previously been described in contexts that suggest a direct involvement in immune escape mechanisms. These include Asp215His, already present in the original isolate and whose prevalence within the population increased in all three experimental conditions (this same amino acid is replaced by a glycine in lineage B.1.351), Gly252Asp, identified in the 30-day isolate of lineage B.1 virus passaged with P40, both experimentally induce by exposure to sub-neutralising concentrations of monoclonal antibodies^19^, and Ser640Phe, identified in the 30-day isolate of lineage B.1 virus passaged with P1280, the latter being identified in a breakthrough infection caused by lineage P.1 in a fully vaccinated man shortly after receiving the second dose of Pfizer BNT162b2 mRNA vaccine (although it is not clear if and to which extent this new mutation contributed)^20^. Other noteworthy mutations on the S gene are Leu141_Tyr144 deletion, identified in the 30-day isolate of lineage B.1 passaged with P40, Tyr145 deletion, identified in the 30-day isolate of lineage B.1 passaged with P160 and considered one of the lineage-defining mutations of lineages B.1.1.7 and B.1.525, and Glu484Ala substitution, identified in the 90-day isolate of lineage B.1 passaged with P40. Deletions of the amino acids Leu141_Tyr144 and Tyr145, have been proven to reposition a glycosylation site on amino acid 149 thus blocking access to the epitope, and reducing susceptibility not only to polyclonal sera, but also to some monoclonal antibodies frequently used in therapy^21,22^. Substitution Glu484Ala, on the other side, alters the RBD local charge from a negative glutamic acid to a nonpolar alanine, thus disrupting non-covalent interactions between the domain and RBD-specific antibodies^22–24^. For further information regarding the specific monoclonal antibodies affected by these mutations and the structural insight into the epitope-antibody interaction, please refer to the cited literature. These three mutations have been identified, by several independent studies, in the sequences of clinical samples of immunocompromised patients with specific B-immunity deficiencies, who often develop chronic SARS-CoV-2 infections with prolonged viral shedding. The longitudinal monitoring of these subjects highlighted a peculiar intra-host viral evolution characterized by the accumulation of an unusually high number of mutations potentially relevant both from a biological and epidemiological point of view, emphasizing how persistent viral replication in the presence of a poorly neutralising immune response or in total absence of immune response may drive the adaptive evolution of SARS-CoV-2^25–31^. A common thread among all these cases is the use, in single or more often repeated administration, of hyperimmune plasma or monoclonal antibodies, which have been proven to shape intra-population genetic diversity causing a massive and dynamic genetic change in the viral population, with the appearance of escape mutations a few days after administration, together with a consistent viral rebound^32^. As evidence of the role of these mutations in immune evasion, the same mutations have been experimentally induced by serial passaging in culture in the presence of sub-neutralising concentrations of monoclonal antibodies. This study demonstrates the possibility of polyclonal convalescent sera to induce the same escape mutations, further addressing the relevance of these epitopes as targets of neutralising antibodies. These mutations have been proven to reduce susceptibility not only to monoclonal antibodies, but also to convalescent sera and, more importantly, to vaccinee sera. The decrease in the neutralising efficacy is significant both for variants bearing Leu141_Tyr144 or Tyr145 deletions and Glu484Ala substitution, but the latter can cause total loss of neutralising power, especially of poorly or moderately neutralising sera, which ultimately results in a complete escape of the virus carrying the mutation^33–35^. Numerous variants that originated independently in different geographical areas, show an aminoacidic substitution at position 484 (lysine in lineages B.1.1.7, P.1, B.1.525, P.2, P.3 and B.1.526, and glycine in lineage B.1.617.1), suggesting that it represents a mutation hotspot particularly subjected to selective pressure which has undergone a convergent adaptive evolution, becoming one of the major contributors to increased resistance to antibody neutralisation shown, to different extent, by these variants^36,37^. Glu484Ala substitution, for its part, was identified in the United Kingdom between April and May 2021 in a small but non negligible number of sequences referable to lineage B.1.617.2^38^. More recently, this substitution was identified in lineage B.1.1.529, together with many other mutations on the spike, which have caused concern in the international scientific community due to their potential for immune evasion from infection- as well as vaccine-induced antibody responses^39,40^. Glu484Ala not only exhibits an escape potential similar to Glu484Lys and Glu484Gln, but some studies also reported an increased infectivity of the variant bearing Glu484Ala substitution in the presence of some convalescent sera, when compared with the same variant cultured in the absence of serum, potentially implying antibody-dependant enhancement mechanisms and suggesting that this mutation could further increase viral fitness in a largely immunised population^33^.

E gene, coding for envelope protein, is also affected by mutations (Ser68Phe in B.1 cultured with P160 and Val5Phe in B.1 cultured with P1280). M gene, on the contrary, is affected by a single mutation, identified in B.1 treated with P40 (Thr7Ile). While all of the mutations identified on the E gene were previously classified as genetic polymorphisms^41^, no information was found for the mutation affecting the M gene, but considering that it falls within the luminal-exposed C-terminal domain of the protein its contribution to immune evasion is unlikely.

In addition, a large number of mutations also affected ORF1ab, thus involving non-structural proteins, which intervene in various ways during viral replication cycle, including nsp2, nsp3, nsp13, nsp14, and nsp15^42^. All the mutations reported have been previously identified in the sequences of clinical isolates, but all have maintained a low and constant prevalence since their appearance, suggesting that they are simply genetic polymorphisms that have no repercussions on viral infectivity. As for mutations on genes coding for accessory proteins, lineage B.1 treated with P40 and lineage B.1 treated with P160 have accumulated two frameshift deletions on ORF7a (Phe101Leufs*5) and ORF7b (Leu14Serfs*17), both resulting in the insertion of a stop codon and the early truncation of the protein (at amino acid positions 105 and 30, respectively, considering canonical lengths of 121 and 43 residues). Similar deletions have seldom been identified in clinical samples, but these variants seem to hardly spread giving rise to community outbreaks, while large-scale transmission events have never been documented, suggesting that such changes have a significant impact on viral infectivity^43,44^. Given the immunomodulatory role of ORF7a and ORF7b as IFN signalling antagonists^45^ and considering that Vero E6 cells do not have an interferon response, these proteins are less subject to constraints in the accumulation of loss-of-function mutations, like those identified in this study. Furthermore, more recent studies not only suggest a structural role for SARS-CoV-2 ORF7b^46^, like for SARS-CoV^47^, a transmembrane protein expressed on the envelope of mature virions, but also document the production of specific anti-ORF7b antibodies during infection^48^ (see below for further consideration regarding their possible involvement in immune evasion).

### Lineages B.1.1.7, B.1.351, P.1 and B.1.525

**Figure 2:**
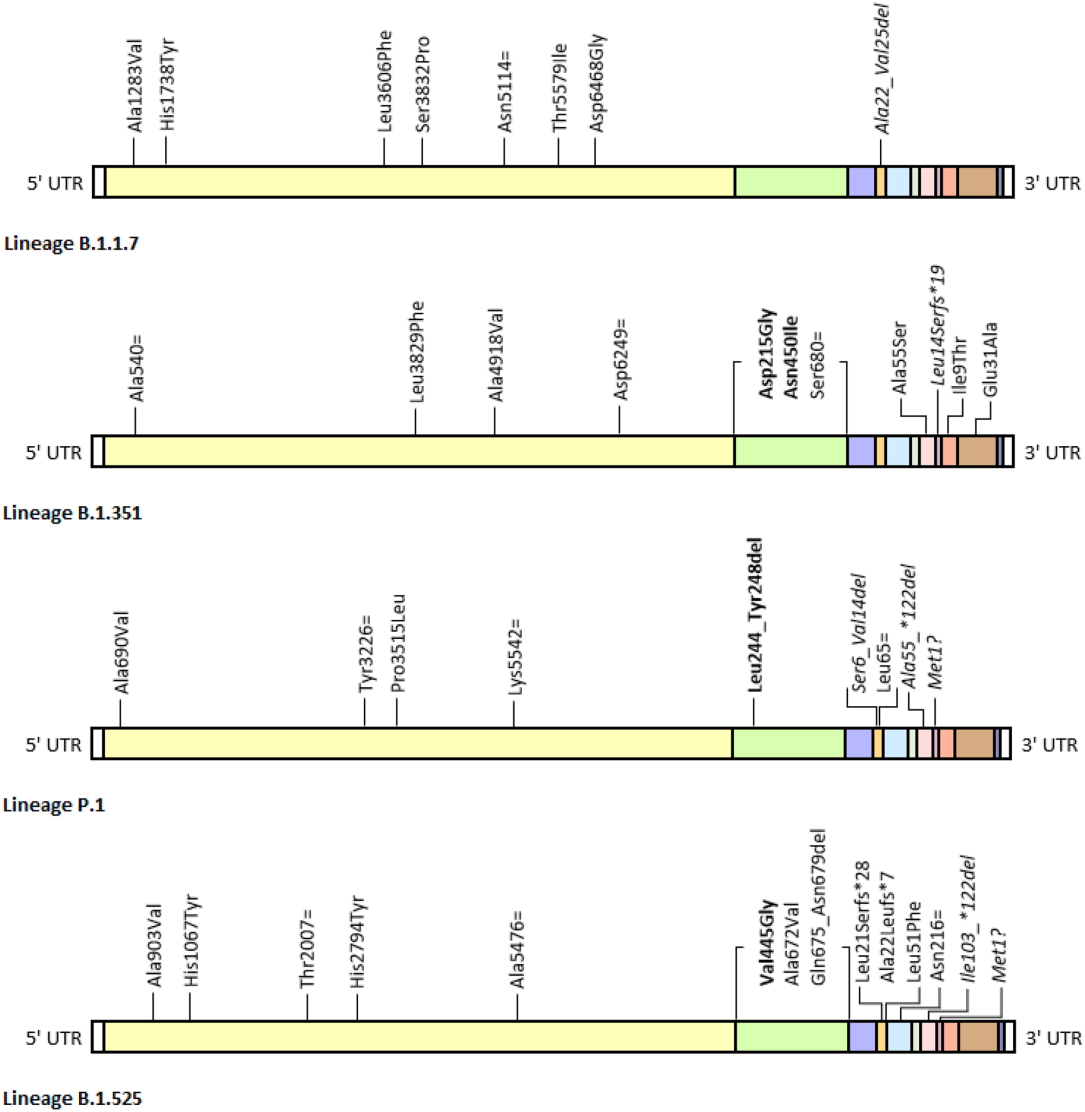
Schematic representation of newly acquired mutations identified in the 90-day isolates of lineage B.1.1.7, B.1.351, P.1 and B.1.525 maintained in culture in the presence of the highly neutralising serum pool (P1280). Known escape mutations are highlighted in bold, while other presumptive escape mutations are highlighted in italics. The “ = ” symbol indicates a synonym mutation, whereas the “fs*” symbol indicates a frameshift mutation causing the early truncation of the protein at the specified amino acidic position. From 5’ UTR to 3’ UTR (both in white): ORF1ab (yellow), S (green), ORF3a (blue), E (orange), M (light blue), ORF6 (grey), ORF7a (pink), ORF7b (lilac), ORF8 (red), N (brown) and ORF10 (lavender).

Compared to lineage B.1, fewer mutations were identified in lineages B.1.1.7, B.1.351 and P.1 over 90 days (9, 12 and 14, respectively), while lineage B.1.525 acquired an exceptionally high number of mutations (25 in total); in any case, the calculated mutation rate was significantly higher than the reference value (9.5·10^–4^, 1.2·10^–3^, 1.5·10^–3^ and 2.6·10^–3^ m/g/year, respectively). Newly acquired mutations identified in the 90-day isolates of lineages B.1.1.7, B.1.351, P.1 and B.1.525 are summarised in Fig. 2.

Similar to lineage B.1, some of the identified mutations fell on ORF1ab, thus affecting the aforementioned nsp2, nsp3, nsp13, nsp14 and nsp15, as well as nsp4 and nsp6. None of these mutations was proven to enhance viral replication in any way.

While in lineage B.1 a considerable number of mutations affected the S gene, in the other lineages mutations involving this gene are much less numerous or totally absent, like in lineage B.1.1.7. Of note, mutations identified in lineages B.1.351, P.1 and B.1.525 are, for the most part, known escape mutations. For lineage B.1.351, these include Asp215Gly substitution, one of the defining mutations for this lineage, not present in the original isolate and later identified in the 60-day isolate sequence, to which a moderate escape potential is attributable, and Asn450Ile substitution, also identified in the 60-day isolate, which falls within the RBD and was proven to confer resistance to some monoclonal antibodies^49,50^. Lineages P.1 and B.1.525, for their part, only accumulated one escape mutation each, that is to say, respectively, Leu244_Tyr248 deletion, identified in the 30-day isolate of lineage P.1, which falls in one of the NTD immunogenic epitopes, is responsible for partial immune escape from convalescent and vaccinee sera, was identified in persistently infected immunocompromised patients^13,27,51^ and is inducible by serial passaging in culture with monoclonal antibodies, and Val445Gly substitution within the RBD, identified in the 30-day isolate of lineage B.1.525, which was also experimentally induced in vitro^33^. Other genetic polymorphisms affecting the S gene include Ser680 = in lineage B.1.351, and Ala672Val and Glu675_Asn679del in lineage B.1.525. All these variants, for their part, accumulated additional mutations in other structural proteins, namely the envelope protein and ORF7b. In lineages B.1.1.7 and P.1, E gene is in fact affected by two inframe deletions, both identified int the 90-day isolates, resulting in loss of amino acids Ala22_Val25 and Ser6_Val14, the first falling within the transmembrane domain and the latter within the luminal-exposed N-terminal domain and the transmembrane domain. On the other hand, deletions on ORF7b coding sequence were identified in lineages B.1.351, P.1 and B.1.525, all resulting either in the truncation of C-terminus or in the complete loss of ORF7b protein. These include a 2-nucleotide frameshift deletion, which results in the insertion of a premature stop codon and the early truncation of the protein at amino acid positions 32, identified in the 90-day isolate of lineage B.1.351 (Leu14Serfs*19), and two much larger deletions (222 and 61 nucleotides, respectively), identified in the 30-day isolate of lineage P.1 (Ala55_*122del, Met1?) and in the 90-day isolate of lineage B.1.525 (Ile103_*122del, Met1?), which, encompassing ORF7a and ORF7b, lead to the synthesis of a truncated fusion protein, unlikely retaining its original functions^52^. Consequences of these mutations on the localization and the antigenicity of envelope and ORF7b proteins have not been determined. Although specific anti-envelope and anti-ORF7b antibodies are produced during infection, it is not clear whether they have a neutralising capacity similar to that of anti-spike antibodies. By virtue of these premises, it cannot be excluded a priori that mutations affecting these proteins can be positively selected by the antibody immune response as escape mutations. However, data supporting this hypothesis are not available so that the actual role played by these mutations in immune escape is unquantifiable. Given the low prevalence, in the human population, of such deletions, it is conceivable that these imply a considerable and non-surmountable fitness disadvantage that overrides the benefit conferred by a hypothetical immune escape capacity, therefore leading to the rapid elimination of these mutations in vivo.

Of note, both for lineage B.1 and other variants, all identified escape mutations, either well-characterised or presumptive, did not emerge in the corresponding untreated control.

### Immune escape

In all lineages a considerable number of characterized escape mutations on the spike, as well as additional putative escape mutations on other structural (envelope proteins) or accessory proteins (ORF7b), were identified. In any case, their emergence and affirmation within the viral population were accompanied by a more or less considerable decline in neutralising power. To quantify the resistance to neutralization acquired by every lineage during co-culturing with neutralising antibodies, pool titres determined against original isolates and 90-day isolates were compared.

For lineage B.1, serum pools neutralization titre decreased by > fourfold for P40, fourfold for P160 and twofold for P1280 during the study, with a nearly total escape from P40, a considerable escape from P160 and a minor escape from P1280.

While in B.1 lineage the escape seems to be primarily attributed to the appearance of mutations on the S gene, in other lineages only escape spike mutations of secondary importance were identified, when present at all, suggesting the involvement of additional mutations in immune escape, specifically those identified on the envelope structural protein and on ORF7b accessory protein. Assuming that these are indeed positively selected escape mutations in the presence of a selective antibody pressure, a hypothesis currently not supported by literature, they appear to have been responsible for a not negligible decrease in the neutralizing titre, in association or not with known escape mutations on the spike, to which, however, according to literature data, it is not possible to entirely attribute this greater resistance to neutralization. In any case, each of these variants showed a significant escape capacity, with a neutralization titre decreased by fourfold in the case of lineages B.1.1.7 and P.1 and twofold in the case of lineage B.1.351 and B.1.525.

## Discussion

Considering the purposes, on the one hand, to estimate to what extent the spread of the infection has contributed to the evolution of B.1 lineage and to the appearance of new variants carrying specific lineage-defining escape mutations and, on the other hand, to evaluate the further ability of these lineages to evolve in response to the pressure exerted by neutralising antibodies, the data obtained allowed to draw interesting conclusions.

For lineage virus B.1, passaging in the presence of a poorly or moderate selective antibody pressure led to the rapid selection of a large number of mutations, including escape mutations, responsible for the acquisition of a partial or total resistance towards the pool of sera used for culturing. The acquisition by lineage B.1 of escape mutations characterizing many of the variants (VOC or VOI) currently in circulation underlines how the genetic barrier that separates this lineage from other variants is relatively low and can be easily overcome in the case of exposure to adequate selective pressures, capable of accelerating the fixation of antigenically and therefore immunologically favourable mutations within the viral population. It also emerged that almost all the escape mutations selected in this study were previously identified in a similar, yet much more complex, context, represented by chronic SARS-CoV-2 infections in immunocompromised subjects, which highlights how these mutations can arise even in vivo in the presence of a sub-optimal antibody response, paving the way for the hypothesis that all the escape mutations currently in circulation in different lineages may have found their origin in the intra-host evolution in an index patient with specific B-immunity deficiencies. These variants, possessing antigenically different characteristics from other variants circulating in the population, do not find obstacles (or find fewer) to their own diffusion, as they are less susceptible to neutralisation by antibodies produced following a previous infection or vaccination. Although it is simplistic to trace the entire genetic diversity of the species back to the evolution within a few immunocompromised individuals and the subsequent spillover in the general population, if we jointly consider the intra-host evolution of potential escape variants in individuals with a deficient immune response and the selection of escape mutations in the immunocompetent population, but which is only rarely able to develop a strongly neutralising antibody response, it becomes clear that each infected individual is actually associated with a risk, more or less great depending on the immune status, to select a variant resistant to the immune response. If we consider the number of people who have contracted the infection, the possibility of seeing a resistant variant emerge becomes a certainty, as demonstrated by the continuous appearance of new variants bearing mutations capable of partially or completely eluding neutralisation by antibodies produced in response to a previous infection or vaccination, thus making the possibility of both reinfection and breakthrough infection, more and more concrete, and questioning the effectiveness of immunisation countermeasures implemented globally to keep the spread of the pandemic under control. Taken together, these data demonstrate the derivation of currently circulating lineages from B.1 and unequivocally identify antibody immune response as one of the driving forces behind the evolution of the species.

As for the other variants, these have accumulated fewer mutations on known targets of the neutralising antibody immune response, namely the spike glycoprotein. Considering that these variants already possess a considerable number of mutations on the S gene (respectively nine, eight, fourteen and eight) and that the same spike, due to its relevance in the viral replication cycle, is subjected to structural and functional constraints that limit the accumulation of mutations, which, while ensuring an escape from the neutralising immune response, could potentially be deleterious for viral infectivity, it is possible that the maximum limit of tolerated mutations on the spike has already been reached or is quite near for all these variants. Alternatively, the reduced number of new spike mutations could be due to the use of convalescent sera collected from subjects who contracted the infection prior to the spread of other variants in Italy, therefore containing specific antibodies for lineage B.1, against which all the considered variants already show some degree of resistance, mainly attributable to the same mutations on the spike. Despite this, the adaptive capacities of these lineages in the presence of a selective antibody pressure cannot be defined as poor, considering the appearance of mutations on the spike glycoprotein as well as on other major or minor structural proteins, such as envelope protein and ORF7b protein, identified in association or not with escape mutations on the spike. These results firmly suggest not only that the role of other proteins as targets of neutralising antibody response has been widely and wrongfully underestimated, but also that mutations falling in their coding sequence may confer resistance to antibody neutralisation, thereby potentially determining immune evasion, similarly to mutations on immunodominant epitopes of the spike glycoprotein.

The identification of new hypothetical escape mutations invites a deeper investigation of virus-immunity interactions to better understand past evolutive pathways and the forces driving this process and, above all, to predict possible future evolutionary trajectories of SARS-CoV-2 within an immunised population. The emergence of new escape mutations outside the spike also encourages to develop broader-spectrum immunisation strategies that do not aim at activating a protective response exclusively against the spike, but which take into account other non-immunodominant but still immunogenic epitopes, therefore guaranteeing greater resistance of the elicited immune responses against the unceasing accumulation of new escape mutations.

Based on these evidences, if, on the one hand, it is logical to hope that the increase in vaccination coverage and the achievement of the much desired herd immunity may limit the spread of SARS-CoV-2, on the other hand, high levels of immunity can constitute a double-edged sword, providing the selective pressures necessary to favour an evolution of the species, hence guaranteeing the persistence in endemic form of SARS-CoV-2 in the population. In the scenario mass vaccination could therefore lead to the end of the pandemic, but the end of the pandemic may not necessarily correspond to the disappearance of SARS-CoV-2, whose continuous evolution will be shaped by dynamic interactions with host immune responses. The identification of the same Glu484Ala substitution in some sequences referable to the Delta variant in the United Kingdom, one of the countries with the highest vaccination coverage, could be a prime (but presumably not the last) example of viral evolution within a largely vaccinated population.

Overall, this study warrants continuous monitoring of SARS-COV-2 evolution, making the implementation of large-scale surveillance initiatives based on SARS-CoV-2 genomic sequencing and their deeper integration into the practices of public health of paramount importance, in order to maximise their impact on infection management, hence allowing a rapid identification of new epidemiologically relevant variants and providing with critical evidence in terms of diagnostic reliability as well as vaccine and therapeutics efficacy.

## Methods

### Cell culture conditions

Vero E6 cell cultures were maintained in Minimum Essential Medium (MEM) supplemented with 2 mM L-glutamine, 100 U/mL penicillin, 100 μg/mL streptomycin (complete culture medium) and 10% heat inactivated foetal bovine serum (FBS)^53–55^. For the preparation of 96-well cell culture plates for viral titration and neutralisation tests, cells were seeded, the day prior to infection, at a density of approximately 20,000 cells per well (2,000,000 cells per plate) using complete culture medium supplemented with 5% of FBS, while for culture infection both in plates, for titrations or for neutralisation tests, and in flasks, for viral propagation and mutational induction assay, complete culture medium supplemented with 2% of FBS was used (infection medium). Cells were incubated at 37 °C in a humidified, 5% CO_2_ atmosphere-enriched chamber until use. Cell culture medium and supplements were purchased from EuroClone (Milan, Italy).

### Viral strain isolation and titration

Viral strains were isolated from positive clinical specimens residual from routine activities, which were submitted to the Unit of Microbiology, Greater Romagna Area Hub Laboratory, Cesena, Italy, for diagnostic purposes and sequenced as part of the project for monitoring the prevalence and distribution of SARS-CoV-2 variants in Italy, promoted by the Italian Institute of Public Health (ISS). Before being used for this study, all samples underwent an anonymization procedure, in order to adhere to the regulations issued by the local Ethical Board (AVR-PPC P09, rev.2; based on Burnett et al.^56^). Both isolation and titration (as well as neutralisation and mutational induction assays, see below) were performed in a BSL-3 laboratory at the Unit of Microbiology, Greater Romagna Area Hub Laboratory, Cesena, Italy. Both the original clinical samples and the viral strains were analysed employing the FilmArray Respiratory Panel (Biomerieux, Marcy l’Etoile, France), testing negative for other respiratory viruses capable of producing cell morphology alterations easily confused with the cytopathic effect caused by SARS-CoV-2. After propagation on Vero E6 cells, each strain was in turn sequenced to reconfirm the lineage identification provided for diagnostic purposes.

Viral strains were titrated using the endpoint dilution method. In brief, serial tenfold dilutions (from 10^–1^ to 10^–10^) in 2% FBS MEM were used to infect confluent monolayers of cells in a 96-well plate, which was afterwards incubated at 37 °C, 5% CO_2_. After 72 h cells were fixed and stained by means of a 4% formaldehyde solution in crystal violet. Absence or presence of cytopathic effect at each dilution, was assessed by comparison of each well with virus control and cell control wells. Viral titres, expressed ad TCID_50_/mL, were calculated with the Reed and Muench formula based on eight replicated for dilution^57,58^. 90-day isolates were titrated using the same protocol.

### Nucleic acid quantification

To evaluate viral replication in cell cultures for the mutational induction assay, the Allplex SARS-CoV-2 Extraction-Free system (Seegene Inc., Seoul, Korea) was used. It consists of a real-time qRT-PCR multiplex assay based on the use of TaqMan probes, which allows simultaneous detection of four target genes, namely E gene, RdRP/S gene and N gene. Sample preparation, reaction setup and analysis were performed accordingly to the manufacturer instructions^59^. Briefly, 15 µL of sample were diluted 1:4 in 45 µL of RNase-free water in a 96-well PCR plate and hence 5 µL of the dilution were transferred to another plate with 16 µL of PCR master mix, containing 5 µL of MOM (MuDT Oligo Mixture, with dNTPs, oligos, primers and TaqMan 5’ fluorophore/3’ Black Hole Quencher probes), 5 µL of enzymes, 5 µL of RNase-free water and 1 µL of internal control for every reaction. A positive and a negative control were included. The assay was run on a CFX96 real-time thermal cycler (Bio-Rad, Feldkirchen, Germany). The amplification process includes cDNA denaturation at 95 °C for 10 s, primers annealing at 60 °C for 15 s and elongation at 72 °C for 10 s (44 cycles). Fluorescent signals were acquired after every amplification cycle. Results analysis and targets quantification were performed with 2019-nCoV viewer from Seegene Inc.

### Convalescent sera and neutralisation test

All the convalescent sera samples used for the mutational induction assay come from residual clinical specimens collected from blood donors in the months between November 2020 and March 2021 as part of the project TSUNAMI (TranSfUsion of coNvalescent plAsma for the early treatment of pneuMonIa due to SARS-CoV-2), promoted by the Italian Institute of Public Health (ISS) and the Italian Medicines Agency (AIFA), aimed at the collection of hyperimmune plasma from voluntary blood donors recovered from COVID-19 as early therapy for patients with SARS-COV-2 pneumonia. For all subjects, any relevant information concerning the course of SARS-CoV-2 infection was obtained, thereby including first and last positive swab dates, symptomatology, hospitalization, and supportive care received. The samples included in this study were conferred to the Unit of Microbiology, Greater Romagna Area Hub Laboratory, Cesena, Italy, for routine diagnostic purposes in order to measure specific anti-S1/S2 IgG levels and determine neutralising efficacy against wild type lineage B.1 SARS-CoV-2. Before being included in this study, all samples underwent an anonymization procedure, in order to adhere to the regulations issued by the local Ethical Board (AVR-PPC P09, rev.2; based on Burnett et al., 2007^56^).

IgG for S1 and S2 subunits of SARS-CoV-2 spike were measured with a commercial chemiluminescence immunoassay (CLIA) kit, LIAISON SARS-CoV-2 S1/S2 IgG (DiaSorin, Vicenza, Italy)^60^. Positive sera with values greater than 40 AU/mL were selected for neutralisation test on cell culture. Sera samples were tested at a starting dilution of 1:10 and then further diluted 1:2 in 2% FBS MEM, reaching a dilution of 1:2560. Each dilution was then mixed with an equal volume of viral solution (final volume: 100 µL per well) at the concentration of 2000 TCID_50_/mL (corresponding to 100 TCID_50_/well). After a 30-min incubation at 37 °C, the mixture was transferred in a 96-well plate containing a sub-confluent Vero E6 cell monolayer. Plates were incubated at 37 °C, 5% CO_2_ for 72 h and then fixed and stained as previously described. Every sample was tested in duplicate. Absence or presence of cytopathic effect at each dilution was assessed by comparison of each well with virus control and no-virus control wells. The neutralisation titre was defined as the reciprocal of the highest serum dilution capable of inhibiting the appearance of a visible cytopathic effect^61^. Given the availability of a small volume of sample, not sufficient to allow the three-month long mutational induction assay to be successfully completed, 8 sera with high neutralising titre (1280), 8 with medium neutralising titre (160) and 15 with low neutralising titre (40) were selected and equal amounts of each sample were pooled together, thus obtaining three serum pools, which were further retested using the same neutralisation protocol to reconfirm their neutralising titre against lineage B.1. For brevity, these three pools were referred to as P1280, P160 and P40. P1280 titre was also tested against lineages B.1.1.7, B.1.351, P.1 and B.1.525. Since these sera were collected from subjects who contracted the infection in a period prior to the increase in the prevalence of the Alpha variant in Italy, which began in early February, when the prevalence was less than 20% (compared to the peak of 88.1% reached soon afterwards, in May 2021), it is plausible that these only contain antibodies produced specifically against lineage B.1. After 90 days of co-culturing, pools were in turn retested with mutated isolates in order to quantify the resistance acquired during the mutational induction assay.

### Mutational induction assay

To select viral variants capable of evading the recognition and neutralisation by antibodies of convalescent subjects, wild type virus was sequentially passaged in Vero E6 cells in the presence of scalar concentrations of serum containing antibodies specific for SARS-CoV-2. Lineage B.1 was maintained separately with low-, medium- and high-neutralising-titre serum pools, whereas Alpha, Beta, Gamma and Eta variants were treated only with the high-neutralising-titre pool.

In the first passage (“passage 1”) five serial two-fold dilutions of the pools were incubated with equal volumes of viral solution at a concentration of 4·10^4^ TCID_50_/mL in a T25 cell culture flask (final volume: 5 mL). Both serum pool dilutions and viral suspension dilutions were prepared in 2% FBS MEM. The selected starting dilutions were different for the three pools: 1:10 for P40, 1:40 for P160 and 1:160 for P1280. After a 30-min incubation at 37 °C, the mixture was transferred to a T25 culture flask containing a sub-confluent monolayer of Vero E6 cells. Cell cultures were incubated for 5 days at 37 °C, 5% CO_2_. For each passage, a cell control flask was included, along with a virus control flask for every viral lineage. Cell controls, on the one hand, helped in detecting the presence or absence of a cytopathic effect in infected cultures in which the virus is co-incubated with neutralising sera. Virus controls, on the other hand, were used to distinguish which of the mutations identified in the treated counterpart are due to adaptation to culture conditions as well as to replication in a non-human cell line and which actually contribute to evasion from antibody neutralisation. In addition to the evaluation of the cytopathic effect after culture fixing and staining, viral replication was also assessed by qRT-PCR. Although it is widely demonstrated that nucleic acid quantification methods can lead to an overestimation of the actual infectious viral titre, they nonetheless represent a viable and more rapid alternative to endpoint titration when a precise calculation of the titre is not strictly required, like in this case^62^. Ct values of virus-serum solutions before their addition to the cell monolayer were compared with Ct values obtained after 5 days of incubation. Each sample was tested in duplicate. The analysis of qRT-PCR data demonstrated the production of a high-titre viral progeny throughout the study, for every lineage (data not shown).

At each passage, for each series of cell cultures (seven series, considering that lineage B.1 was treated separately with the three pools), the culture with the lowest dilution of serum showing a complete cytopathic effect was selected to be further passaged, hence promoting the replication of viral populations that are capable to replicate at sub-neutralising concentrations of antibodies, in order to mimic the possible selection SARS-CoV-2 underwent in vivo in subjects with suboptimal antibody responses.

For the following passages (“passages 2–18”) the supernatant of the identified culture was subsequently diluted and re-incubated with scalar serum dilutions. In the first passages, dilutions of the supernatant were progressively increased from 1:50 to 1:100, 1:250, 1:10,000, considered too high, and then finally standardized to 1:1000 in order to reach an adequate concentration of the viral inoculum, low enough not to immediately saturate the neutralising antibodies present in the solution and not to deplete susceptible cells, but at the same time sufficiently high to allow multiple cycles of replication in the presence of an inhibitor. Consider that the virus dilution is calculated on the final volume of 5 mL per flask. As for the serum pools, the starting dilution was adapted during the course of the assay, independently for every lineage, choosing to use a lower dilution in the case of the acquisition of a resistance against neutralisation.

Isolates corresponding to 30, 60 and 90 days of culture in the presence of sera (passages 6, 12 and 18) were subjected to whole genome sequencing. As for virus controls, only the 90-day isolates were sequenced. Considering the high viral load of the isolates, approximately inferable from qRT-PCR data after five days of culture (Ct values never exceeded 20, data not shown), it was not necessary to propagate them before sequencing.

### Viral RNA extraction, library preparation and sequencing

To isolate the viral genetic material for sequencing an RNA extraction and purification step was performed using the Maelstrom 9600 system (TANBead—Taiwan Advanced Nanotech Inc., Taiwan). Library preparation was performed using the CleanPlex SARS-CoV-2 Flex Research and Surveillance NGS Panel (Paragon Genomics, Inc., Hayward, CA, USA). All the protocol steps (viral RNA reverse transcription, multiplex PCR, digestions and indexing PCR) were performed according to the manufacturer’s instructions^63^ using the two-pool workflow for multiplex amplification and i7 and i5 indexes for Illumina for final indexing. The entire process was performed using the Microlab STAR automated workstation (Hamilton, Reno, NV, USA). Libraries were then quantified using a Qubit Fluorometer with the dsDNA HS Assay Kit (Thermo Fisher Scientific, Waltham, MA, USA), normalized to 10 nM, pooled in equimolar ratios to reach the recommended final concentration of 4 nM following the Standard Normalization protocol on MiSeq System Denature and Dilute Libraries Guide (Illumina, San Diego, CA, USA), and finally denatured and diluted to 1 pM. Paired-end and dual-indexed sequencing was carried out on a MiSeq instrument (Illumina), with reagent kit v2, using 5% of 10 pM spike-in PhiX as control for low diversity libraries. Sequenced reads were aligned and compared with the reference genomic sequence of SARS-CoV-2 Wuhan-Hu-1 isolate (Access: NC_045512, Version: NC_045512.2) using SOPHiA DDM platform software (SOPHiA Genetics, Lausanne, Switzerland), for determination of the consensus sequence, variant calling and lineage assignment. The mutational induction assay was carried out once. We recognise that performing the study only once might imply uncertainties regarding some of the identified mutations, but the main purpose of our study was to elucidate SARS-CoV-2 mutational and consequently adaptive potential when exposed to a selective pressure, represented, in our case, by a sub-inhibitory concentration of neutralising antibodies. On the one hand, our results allowed us to partially recapitulate SARS-CoV-2 evolution during the pandemic; in this case, the acquisition, by the cultured ancestral virus (lineage B.1), of some of the mutations characterising other lineages confirms the validity of our results. Moreover, the fact that some of the identified mutations had previously arisen in a similar but yet much more complex contexts, represented by chronic infections in immunocompromised patients, further corroborates our findings. On the other hand, we admit the lack of validation for the putative escape mutations we identified in the envelope and ORF7b proteins coding sequence, which had never been characterised as targets of the neutralising immune response. Although potentially representing a limitation of our study, these results might also be one of its strengths, suggesting future potential SARS-CoV-2 adaptive strategies. Our results concerning these other structural proteins certainly represent a novelty and need further confirmation, constituting a breeding ground for future research. Additionally, we believe that performing the mutational induction assay in duplicate would have potentially increased the risk of cross-contaminations between the two separate replicates or between different lineages of the same replicate, thus representing an additional variability source and consequently a potential confounding factor.

The study was conducted according to the guidelines of the Declaration of Helsinki, and approved by the Institutional Review Board of AUSL Romagna (protocol code “COVdPCR” of 07/02/2020).

### Informed consent

Informed consent was obtained from all patients involved in the study.

## Data Availability

Original isolates sequences are available on GISAID. Their accession codes are listed below: EPI_ISL_1908157 (lineage B.1), EPI_ISL_7313989 (lineage B.1.1.7), EPI_ISL_7313911 (lineage B.1.351), EPI_ISL_7313855 (lineage P.1), EPI_ISL_7313934 (lineage B.1.525). Every escape mutation emerged in viruses co-cultured with neutralising sera which was considered relevant for the purposes of this work is discussed in the main text and depicted in figures. Mutations characterized as genetic polymorphisms were not discussed in depth in the Result section, but they were nonetheless included in figures for completeness. As such, all data supporting the findings of this study are either present within the article or accessible through GISAID. In the interest of providing fuller information, complete sequences of treated viruses as well as of untreated controls are available from the corresponding author, upon reasonable request.
